# Burkitt Lymphoma of the Appendix Presenting With Acute Appendicitis and Acute Kidney Injury: A Case Report and Review of Literature

**DOI:** 10.7759/cureus.28392

**Published:** 2022-08-25

**Authors:** Hafiz A Yahya, Vijay Kumar, John T Lam

**Affiliations:** 1 Pathology, University of Mississippi Medical Center, Jackson, USA; 2 Hematopathology, University of Mississippi Medical Center, Jackson, USA

**Keywords:** lymphoma, acute appendicitis, acute kidney injury, burkitt lymphoma, appendix

## Abstract

The gastrointestinal tract is one of the most common sites for extranodal Burkitt lymphomas (BLs), but an appendiceal BL is extremely rare. We describe a case of appendiceal BL presenting with acute appendicitis and acute kidney injury. A 15-year-old obese male presented to the emergency department with fever and right-sided abdominal pain. WBC count was slightly increased, and a CT scan of the abdomen showed features of ruptured appendicitis and peritonitis. The patient was placed on antibiotics, and an interval appendectomy was planned. The patient developed a worsening acute kidney injury one day later, requiring a laparoscopic appendectomy. Gross examination of the appendix revealed a dilated, firm, sausage-like appendix with a hemorrhagic serosa and a firm mesoappendix. Microscopic examination of the appendix showed a dense diffuse infiltration of monomorphic medium-sized atypical lymphoid cells with round nuclei, dispersed chromatin, and small nucleoli. Few scattered macrophages created a vague "starry sky" appearance. Many mitotic figures were seen. The lesion also involved the mesoappendix. Immunohistochemical analysis showed that the lymphoma expressed CD10, CD20, and BCL6 but was negative for CD34, BCL2, and TdT. Later, the fluorescence in situ hybridization (FISH) analysis detected an IGH-MYC (8;14) fusion. A final diagnosis of appendicular Burkitt lymphoma was made. Two weeks later, a bone marrow biopsy performed for staging showed involvement of bone marrow by BL. The patient lost follow-up after that due to the transfer of care to another hospital.

## Introduction

Burkitt lymphoma (BL) is an aggressive B-cell lymphoma first described in 1958 by Dennis Burkitt as sarcoma afflicting the jaws of African children [1]. Interestingly, Epstein-Barr (EBV) was later discovered in the culture of these tumor cells [2]. BL is predominantly seen in children and immunocompromised patients and is associated with 8;14 (q24;q32) translocation [1].

Although BL is considered a nodal lymphoma, it most commonly presents as an extranodal disease. The gastrointestinal tract and head and neck are the most prevalent sites for extranodal BL. Bone marrow, genitourinary tract, central nervous system, and liver are less common sites. Current therapies allow for long-term survival in most patients and even cures in some cases of BL. Poor prognostic indicators include age >40 years, Eastern Cooperative Oncology Group (ECOG) performance status ≥2, serum lactate dehydrogenase (LDH) >3X upper limit of normal, bone marrow and central nervous system involvement, and a delay in diagnosis [3-5].

Appendiceal Burkitt’s lymphoma is extremely rare; we found <20 reports published to date. This paper presents a rare case of BL of the appendix diagnosed in a child with an unusual presentation of acute appendicitis and acute kidney injury.

## Case presentation

A 15-year-old obese male presented to the emergency department with right-sided abdominal pain, constipation, tenesmus, dysuria, nausea, and vomiting for one week. He had no history of fever, hematuria, blood in stool, weight loss, penile discharge, or kidney stones. Physical examination showed a diaphoretic morbidly obese male (BMI: 40.19 kg/m²) with tenderness on the right side of the abdomen. No guarding or rigidity was noted. Bowel sounds were normal. Laboratory investigations are shown in Table 1.

**Table 1:** Laboratory investigations μl: microliter; mm^3^: cubic millimeter; g/dL: grams per deciliter; mg/dL: milligrams per deciliter; U/L: units per liter

Component (unit)	Patient	Reference Range
White blood cell count (per *μ*l)	11500	3800 - 9800
Red blood cell count (million/mm3)	3.35	4.03 - 5.29
Hemoglobin (g/dL)	9.9	11.0 - 14.5
Hematocrit (%)	39.8	33.9 - 43.5
Platelet count (per *μ*l)	336,000	175,000 - 332,000
Total bilirubin (mg/dL)	0.35	0.15 - 1.00
Alanine aminotransferase (U/L)	23	0 - 41
Aspartate aminotransferase (U/L)	21	0 - 40
Alkaline phosphatase (U/L)	70	74 - 390
Serum lipase (U/L)	16	13 - 60
Urine creatinine (mg/dL)	77	40 - 278
Serum creatinine (mg/dL)	1.6	0.2-0.7

**Figure 1:**
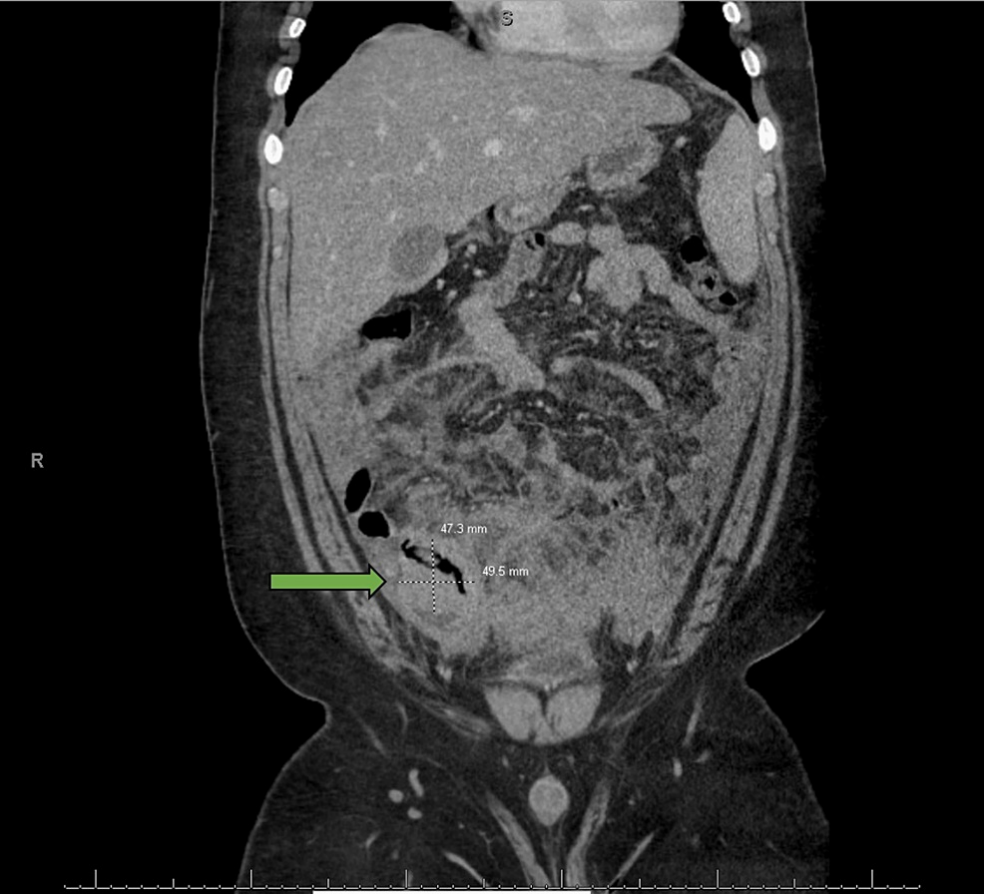
Computerized tomography (CT) scan of the abdomen CT scan of the abdomen showed extensive inflammatory stranding in the lower abdomen and pelvis centered in the right lower quadrant and a thick-walled fluid collection (as indicated by the arrow) in the pelvis concerning for ruptured appendicitis.

An abdominal computed tomography (CT) scan showed extensive inflammatory stranding/phlegmon in the lower abdomen and pelvis, centered at the right lower quadrant. A thick-walled fluid collection (4.7 x 4.9 cm) in the pelvis concerning for ruptured appendicitis was noted (Figure 1). Peritoneal stranding/thickening concerning for peritonitis was also seen. Right hydronephrosis and hydroureter were also seen. Based on the symptoms and CT findings, he was placed on IV antibiotics and an interval appendectomy was planned.

A day later, the patient developed a worsening acute kidney injury (serum creatinine raised to 2.86 mg/dL (N: 0.2-0.7 mg/dL)). He underwent cystoscopy with bilateral retrograde pyelogram ureteral stent placement. Creatinine improved slightly following the procedure. A decision was made to take the patient to the operation theater for a laparoscopic appendectomy and to perform a kidney biopsy due to suspicion of acute interstitial nephritis as a cause of acute renal failure.

**Figure 2:**
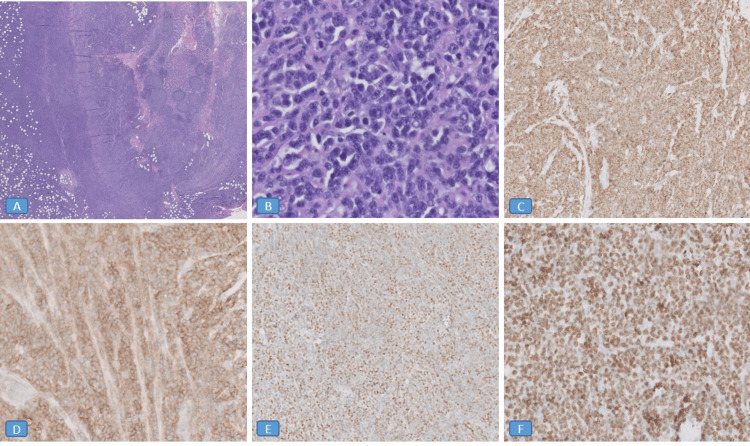
Burkitt lymphoma of the appendix Microscopic examination showed a dense diffuse infiltrate of monomorphic medium-sized atypical lymphoid cells with round nuclei, dispersed chromatin, and small nucleoli. Few scattered macrophages created a vague "starry sky" appearance. Many mitotic figures were seen. The infiltrate also involved the mesoappendix (Figures 2A, 2B). Immunohistochemical stains showed that the atypical lymphoid cells were positive for CD79a (Figure 2C), CD10 (Figure 2D), BCL6 (Figure 2E), and Ki-67 (expressed in 100% of lymphoma cells) (Figure 2F). Figures 2A, 2E were taken at 2X magnification. Figure 2B was taken at 40X magnification. Figures 2C, 2D, 2F were taken at 20X magnification. CD: cluster of differentiation

During surgery, the appendix was significantly inflamed with phlegmon formation. No macroscopic perforation or necrosis was noted. Inspection of the terminal ileum revealed significant dilation and inflammation with creeping fat. Gross examination of the specimen showed a dilated, firm sausage-like appendix measuring 9.5 x 0.8 x 0.7 cm with a hemorrhagic serosal surface and an attached firm mesoappendix (6.2 x 2.0 x 2.1 cm). A cross-section of the specimen did not reveal any definitive mass in the appendiceal lumen. Microscopic examination, as shown in Figure 2, revealed a dense diffuse infiltrate of monomorphic medium-sized atypical lymphoid cells with round nuclei, dispersed chromatin, and small nucleoli. Few scattered macrophages created a vague "starry sky" appearance. Many mitotic figures were seen. The infiltrate also involved the mesoappendix (Figures 2A, 2B). Immunohistochemical stains showed that the atypical lymphoid cells were positive for CD20, CD79a (Figure 2C), CD10 (Figure 2D), BCL6 (Figure 2E), CD45, and Ki-67 (expressed in 100% of lymphoma cells) (Figure 2F); and negative for CD3, CD34, TdT, BCL-2, EBER, CAM 5.2, synaptophysin, and chromogranin.

Flow cytometry was not performed on the appendix, as the specimen was received in formalin. Two weeks later, a bone marrow biopsy performed for staging showed atypical lymphoid cells comprising approximately 30% of the marrow area. Flow cytometry performed on bone marrow showed a CD10+ B-cell lymphoma with kappa light chain restriction. Overall findings, including immunohistochemical analysis, were consistent with the involvement of lymphoma. Later, FISH analysis performed on the appendix detected an immunoglobulin heavy-chain (IGH)-MYC (8;14) fusion and loss of BCL2. A final diagnosis of appendicular Burkitt lymphoma was made. Hematology/Oncology was consulted to initiate chemotherapy, but the patient lost follow-up due to the transfer of care to a hospital near the family's residence.

## Discussion

Gastrointestinal (GI) lymphomas account for 4-20% of non-Hodgkin's lymphomas and 30-45% of extranodal lymphomas [3]. Burkitt lymphoma is an aggressive non-Hodgkin B-cell lymphoma [4,5]. The stomach is the most commonly involved organ, followed by the small intestine, pharynx, and large intestine. The disease is more common in males. Children and immunocompromised patients are most often affected [6].

Acute appendicitis is a characteristic of most appendicular tumors, including lymphomas [7,8]. Patients with Burkitt lymphoma of the appendix often present at an earlier age (33 years) than those with follicular lymphoma (59 years) or diffuse large B-cell lymphoma (53 years), as occurred in our case.

There are no classical imaging features of appendiceal lymphomas. Diffuse enlargement of the appendix with thickening of its wall and periappendiceal tissue can be seen on imaging, representing a direct serosal extension of lymphomatous cells or a related inflammatory process. The diameter and size of the lymphomatous appendix on CT scan are usually >3 cm, which is larger than the expected size of non-tumor appendicitis [9-12]. However, in our case, the CT scan revealed no abdominal lymphadenopathy, and the appendix was not clearly visible. The diagnosis of Burkitt Lymphoma was only made after histopathological examination.

Our case was unusual in that it presented with acute appendicitis and acute renal failure mimicking acute interstitial nephritis. Besides, our patient was kept on antibiotics for a week to treat appendicitis conservatively, increasing the chances of a poor outcome due to a delay in diagnosis of Burkitt lymphoma. In addition to bone marrow and CNS involvement, poor prognostic indicators of BL include a delayed diagnosis.

We want to highlight that the risk associated with delayed lymphoma diagnosis must be considered when examining the risks and benefits of operative versus nonoperative management of acute appendicitis. Moreover, we want to reiterate that all appendectomy specimens should undergo a histological evaluation to avoid missing potential deadly diseases.

## Conclusions

Primary Burkitt lymphoma of the appendix is extremely rare. Appendicitis is one of the most frequent presentations of primary appendiceal lymphoma. Therefore, all appendectomies must undergo a histopathological examination. When comparing the risks and benefits of operative versus nonoperative management of acute appendicitis, it is important to consider the potential risk of late diagnosis of lymphoma in patients with appendicitis undergoing conservative management.
